# Does air quality improvement promote enterprise productivity increase? Based on the spatial spillover effect of 242 cities in China

**DOI:** 10.3389/fpubh.2022.1050971

**Published:** 2022-11-25

**Authors:** Dong Le, Yusong Li, Fei Ren

**Affiliations:** School of Economics, Zhongnan University of Economics and Law, Wuhan, China

**Keywords:** air quality improvement, spatial spillover effect, enterprise productivity, enterprise innovation quality, human capital, resident health

## Abstract

**Introduction:**

Air pollution not only harms people's health, but also impedes urban economic development. This study aims to analyze how air quality improvement affects enterprise productivity. And then from regional and time heterogeneities' aspects to investigate if the air quality improvement increase enterprise productivity.

**Methods:**

The data were obtained from China Industrial Enterprise Database and China Patent Database,and this study used Spatial Durbin Model to analyze how air quality improvement affects enterprise productivity.

**Results:**

The results show that: (1) air quality improvement and its spatial spillover effect can significantly increase enterprise productivity in adjacent areas. (2) After 2010, the government implemented more stringent measures to prevent and control air pollution, which made the air quality improvement promote enterprise productivity increase more obviously. The air quality improvement in eastern and central regions was less obvious than in western regions. (3) Air quality improvement can increase enterprise productivity by improving enterprise innovation quality, ensuring the health of urban residents, and increasing the stock of urban human capital.

**Conclusion:**

Air quality improvement and its spatial spillover effect can significantly increase enterprise productivity in adjacent areas. So this study puts forward some policy enlightenment, such as establishing an air pollution detection system, using an intelligent network supervision platform, and implementing a coordinated defense and governance system.

## Introduction

According to the report on China's Ecological and Environmental Conditions in 2020, only 201 cities in China have reached the air standards, accounting for 59.9% of the total number of cities. Additionally, air pollution causes 1.2 million to 1.4 million deaths every year (1, 2), and the direct and indirect economic losses account for 1 to 8% of China's GDP. Serious air pollution causes huge losses to the economy and harms people's health (3, 4). Under the policy background of “Lucid waters and lush mountains are gold and silver mountains,” reasonable and effective measures to improve air quality have become the key to promoting the development of enterprises and ensuring the physical health of residents. The Party Central Committee also requires provinces and cities to strengthen comprehensive measures to win the battle against air pollution. However, the local government in the development of the regional economy ignores air protection, especially in China's western region, where excessive emissions, low efficiency of energy utilization, and low awareness of environmental protection consciousness exist. In air quality improvement, the local governments of neighboring cities are buck-passing each other, which makes the air quality improvement measures of the central government fail to be fully implemented (5). So, does the improvement of air quality and economic development are mutual contradiction? The in-depth study of these problems will provide an important theoretical basis and practical significance for China's high-quality economic development.

In recent years, scholars have carried out a study on the economic benefits of air quality improvement, and the research shows that air quality improvement will promote enterprise productivity increase. In existing studies, air quality improvement on enterprise productivity is mainly focused on the individual micro field, a fixed enterprise, or an industry, such as pear packaging factory workers (6), athletes (7), textile workers (8), Ctrip employees (9), and prisoners (10). Additionally Zhou and Li (11) found that air pollution significantly worsens income distribution in China, they also proved that increasing health expenditures and declining labor productivity mediate the effect of air pollution on income distribution. Through research, He and Ji (12) found that the cause of enterprise productivity loss was the impact on the health of employees. Moreover, when employees were exposed to more PM2.5, the physical activity index decreased significantly and the disease prevalence increased. Farzanegan et al. (13) found that air pollution has a positive and significant effect on outmigration. They also propose air pollution harmfully impacts the physical and mental health of citizens, reducing enterprise productivity and student academic performance. As the micro individual's enterprise productivity is easy to measure. However, owing to the narrow sample selection range, the air quality improvement has little impact on enterprise productivity. Additionally, the existing research neglected the spillover effects, making its conclusion not reflect real economic laws.

The marginal contribution of this study mainly includes three points: First, the significant spatial spillover effects of air pollution have been ignored in the existing literature. Air quality improvement will affect the productivity of enterprises in the local area. Air quality improvement reduces pollution emissions from local areas while reducing the number of pollutants scattered through air flowing to adjacent areas. Finally, the negative effects of air pollution on the production of enterprises and the health of residents in adjacent areas are reduced. Therefore, the spatial spillover effect cannot be ignored in the study of air pollution. This study used the Spatial Durbin Model to examine the spatial spillover effect and transmission mechanism of air quality improvement to enterprise productivity. Second, in the existing study, almost all choose individual microdata to measure enterprise productivity. These measuring defects make the study's results not universal to a certain extent. This article uses China Industry Business Performance Data to measure enterprise productivity, covering more than 90% of China's enterprises. This kind of measurement could ensure the study's conclusion has a certain universality. Third, existing literature often ignores the endogeneity problem in the model when investigating the impact of air quality improvement on enterprise productivity. This results in a deviation from the real situation. This study uses airflow coefficient, and frequency of environmental protection words in government reports as IV variables to relieve the endogenous problems of the model.

## Theoretical analysis

If air quality improvement, the local government introduces green production technology, ensuring the stock of human capital and residents' health to promote enterprise productivity increase. Additionally, the reduction of pollution emissions from adjacent areas could reduce the negative impact on the health of residents and enterprises' production. Therefore, this section analyzes the impact of air quality improvement on enterprise productivity from spatial spillover effect, enterprise innovation quality, human capital, and residents' health.

### Air quality improvement promotes enterprise productivity increase through the spatial spillover effect

When the air quality of the adjacent area is improved, the emission of air pollutants in the neighboring area is reduced. Conversely, air pollutants have natural features of drifting with the atmosphere, so when the air quality is improved, the adjacent areas scatter fewer air pollutants into the local area. Thus, the local area of the air pollutants will be reduced. So local areas' enterprise production and residents' health will be guaranteed. In contrast, adjacent areas' air quality improvement forces enterprises to actively develop green production technology and bring in advanced management experience. The green technology and advanced management experience have positive externalities, so they can spill over to local areas to promote the local areas' enterprise productivity increase.

### The improvement of air quality forces enterprises to enhance the quality of innovation and promote enterprise productivity increase

#### The improvement of air quality forces enterprises to strengthen technological innovation and promote enterprise productivity increase

When an enterprise produces under unregulated and unrestrained conditions, the increase in output can be realized by increasing enterprise input. This atmosphere is used to absorb emissions which suppress enterprises from developing innovative technology. However, in the development of the economy, the government also requires the enterprise to reduce pollution emissions. The government forces enterprises to strengthen green innovation technology (14, 15). This is because advanced green technology can optimize the allocation of resources and reduce the undesirable output in enterprise production. Green technology also could make capital, information, and technology transfer from traditional labor-intensive industries to the technology-intensive enterprise. The technology-intensive enterprises can increase returns to scale and decrease overflow cost, drive the upstream and downstream affiliated enterprise development innovatively, the city enterprise innovation quality levels increase, and ultimately promote enterprise productivity.

#### Air quality improvement forces enterprises to improve energy efficiency and promote enterprise productivity increase

Improving energy utilization efficiency is a necessary measure of air quality improvement. China's energy mainly comes from coal and oil. According to the successful experience of air pollution improvement in developed countries, it is particularly important to improve the energy efficiency of fossil fuels to improve air quality. The improvement in the utilization efficiency of coal, oil, and other fossil fuels is mainly reflected in processing, combustion, gasification, liquefaction, and desulfurization. However, there is still a big gap between China and developed countries in terms of coal chemical technology, coal conversion, desulfurization, denitration, efficient dust removal, and other technologies. The improvement of enterprises' green technology innovation level will force enterprises to optimize the industrial structure, improve the fossil energy used efficiency, and reduce the emission of pollutants from enterprises. Finally, advanced green technology improves the air quality increase effectively.

### Improving air quality will increase the stock of human capital and promote enterprise productivity increase

Human capital is the driving force of urban enterprise productivity improvement (6, 16). The larger the inflow of urban labor, the more is human capital stock (17). Since air pollution can reduce workers' wages and quality of life, the more livable the air environment in a city is, the more residents' intention to move out will be greatly reduced (18). Conversely, the improvement of air quality attracts labor inflow and increases the stock of urban human capital (19). It ensures the technological and educational human capital needed for enterprise productivity improvement. In contrast, air pollution reduces productivity by causing premature deaths of employees. Additionally, it will lead to a higher incidence of diseases, increasing the economic burden on enterprises and individuals. The economic loss caused by the premature death of employees can be estimated by the value of life method (20). The estimation formula is as follows:


(1) $$\begin{array}{l} DED=\sum_{i}1.68\times \frac{CDI_{i}}{CDI_{B}}\times EM_{i} \end{array}$$

where DED is the economic loss caused by the premature death of employees, CDI_*i*_ stands for per capita disposable income in REGION *i*, CDIB is per capita disposable income in Beijing, and EM_*i*_ represents the number of premature deaths due to air pollution in the city *i*.

The loss resulting from increased morbidity of laborious diseases can be measured by Disease Cost Method. The estimation formula is as follows:


(2) $$\begin{array}{l} DEI=\sum_{ij}HE_{ij}=\sum_{ij}E_{ij}\times RP_{ij}+\frac{t_{i}}{2\text{4}2}\times GDP_{i} \end{array}$$

(3) $$\begin{array}{l} RP_{ij}=RP_{zj}*\frac{CDI_{i}}{CDI_{Z}} \end{array}$$

(4) $$\begin{array}{l} TEL=DED+DEI \end{array}$$

DEI is the financial burden of disease, *j* is the type of disease caused by air pollution, *j* represents region, and *HE*_*ij*_ is the economic burden caused by category j diseases in region *i*. *RP*_*ij*_ represents the outpatient fee, or hospitalization fee caused by class *j* diseases in city *i*. *RP*_*zj*_ represents the overall outpatient fee, or hospitalization fee of *j* diseases in Province *Z*. *E*_*ij*_ represents the number of residents with j diseases caused by air pollution in Region *i*. *TEL* stands for direct economic losses caused by air pollution (21). The improved air quality will reduce the incidence of respiratory diseases, hospitalizations, and deaths among residents (22, 23). It ensures healthy human capital for enterprise productivity improvement. Human capital increases the agglomeration effect of the production factor. It also promotes the transfer of production factors from low value-added processing industry to high value-added high-technology industry, promoting enterprise productivity increase.

## Research methods

### Model specification

Air quality improvement can reduce the number of air pollutants scattered into the adjacent area through air circulation. Therefore, the spatial spillover effect must be considered in the study of air quality improvement. The influence of different spatial model express different mechanisms, SEM model assumes that the air quality improvement's effect mainly through the error term to affect, SLM model assumes that the enterprise productivity is mainly through the space interaction effects to change other cities' enterprise productivity, while the SAC and SDM model takes into account of the error term and the enterprise productivity's spatial spillover effects. In addition, the SDM model also considers the influence of spatial interaction, that is, the improvement of enterprise productivity in local areas is not only affected by the local areas' air quality improvement, but also affected by the adjacent areas' air quality improvement. In this study, the LR test is implement on the model, and the results show that the corresponding *p*-value of λ^2^ is <0.1, which indicates that the spatial model of SLM, SEM, and SAC cannot replace SDM. So the Spatial Durbin Model (SDM) was used to study the impact of air quality improvement on enterprise productivity. This model considers the spatial interaction effect of air quality improvement in adjacent areas when studying the effect of air quality improvement on enterprise productivity (24). The formula of the model is as follows:


$$\begin{array}{l} TFP_{it}=\beta_{0}+\delta W\cdot TFP_{it}+\beta_{1}\cdot \ln\;AQI_{it}+\beta_{2}W\cdot \ln\;AQI_{it} \end{array}$$

(5) $$\begin{array}{l} \;+\beta_{3}W\cdot X_{control}+\beta_{4}X_{control}+\mu_{it} \end{array}$$

*TFP* is enterprise productivity, ln *AQI* s air quality improvement, μ_*it*_ is a disturbing term, *W* is spatial weight matrix, and δ*W*·*TFP* and β_2_*W*·ln *AQI*, respectively, express the spatial influence on *TFP* and ln *AQI*. If the value δ is significant and not zero, the coefficient of *W*·*TFP*, *W*·ln *AQI*, and ln *AQI* is greatly different from the traditional regression coefficient. Lesage and Pace found that the partial differential method could solve this deviation (25). Therefore, partial differentiation of the spatial regression model is carried out in this study, the equation is as follows:

Convert the general form of the SDM model into:


(6) $$\begin{array}{l} (I_{n}-\delta W)Y=\iota_{n}\beta_{0}^{'}+\beta X+\theta WX+\varepsilon \end{array}$$

Order, $P\left(W\right)={\left(I_{n}-\delta W\right)}^{-1},Q_{m}\left(W\right)\cdot \left(I_{n}\beta_{m}+\theta_{m}W\right)$, then formula (6) can be converted to formula (7),


(7) $$\begin{array}{l} Y=\sum_{m=1}^{k}Q_{m}(W)X_{m}+P(W)\iota_{n}\beta_{0}^{'}+P(W)\varepsilon \end{array}$$

The following expression can be obtained by transforming Equation (6) into matrix form:


$$\begin{array}{l} \left[\begin{array}{c} Y_{1}\\ Y_{2}\\ Y_{3}\\ \vdots \\ \vdots \\ Y_{n} \end{array}\right]=\sum_{m=1}^{k}\\ \left[\begin{array}{ccccc} Q_{m}{\left(W\right)}_{11} & Q_{m}{\left(W\right)}_{12} & \cdots & \cdots & Q_{m}{\left(W\right)}_{1n}\\ Q_{m}{\left(W\right)}_{21} & Q_{m}{\left(W\right)}_{22} & \cdots & \cdots & Q_{m}{\left(W\right)}_{2n}\\ \vdots & & \ddots & & \vdots \\ Q_{m}{\left(W\right)}_{(n-1)1} & Q_{m}{\left(W\right)}_{(n-1)2} & & \ddots & Q_{m}{\left(W\right)}_{(n-1)n}\\ Q_{m}{\left(W\right)}_{n1} & Q_{m}{\left(W\right)}_{n2} & \cdots & \cdots & Q_{m}{\left(W\right)}_{nn} \end{array}\right]\\ \left[\begin{array}{c} X_{1m}\\ X_{2m}\\ X_{3m}\\ \vdots \\ \vdots \\ X_{nm} \end{array}\right]+P(W)(\tau_{n}{\beta^{\prime }}_{0}+\varepsilon ) \end{array}$$

M = 1, 2, 3… K represents m explanatory variables. The elements on the diagonal of the formula matrix reflect the impact of the local area's air quality improvement on the local area's enterprises' productivity; it is a direct effect. The non-diagonal elements in the matrix reflect the impact of the adjacent area's air quality improvement on the local area's enterprises' productivity; it is an indirect effect, also known as the spatial spillover effect. The sum of direct and indirect effects is total utility. Direct effect: $direct=\frac{\partial Y_{i}}{\partial X_{im}}=Q_{m}{\left(W\right)}_{ii}$, Indirect effect: $indirect=\frac{\partial Y_{i}}{\partial X_{jm}}=Q_{m}{\left(W\right)}_{ij}$, Total effect:*total* = *Q*_*m*_(*W*)_*ii*_ + *Q*_*m*_(*W*)_*ij*_.

This study examines the effect of air quality improvement on enterprise productivity, and the spatial spillover effect of air quality improvement in adjacent areas is obvious. Therefore, the adjacent spatial matrix is used for the model, and the elements of the adjacent matrix are constructed as follows:


(8) $$W_{ij}=\left\{ \begin{array}{l} \begin{array}{cc} 1, & city\;i\;and\;city\;j\;adjacent \end{array}\\ \text{0, }others \end{array}\right.$$

Considering that distance between cities is not the only factor affecting the spatial spillover effect, regional economic disparities also affect the degree of improvement in air quality. Therefore, this study uses per capita GDP to construct the weight matrix of economic distance. The economic distance matrix is constructed as follows:


(9) $$W_{ij}=\{\begin{array}{l} \frac{1}{\left|\bar{Y_{i}}-\bar{Y_{j}}\right|},i\neq j\\ 0,i=j \end{array}$$

And $\bar{Y_{i}}=\frac{1}{T-T_{0}}\sum_{T=t_{0}}^{T}Y_{it}$, Y is the GDP, $\bar{Y}$ is the average GDP, and T is the year.

### Spatial correlation test

Moran's I is used to test the spatial correlation (26) to investigate the spatial effect of air quality improvement; the formula is as follows:


$$\begin{array}{l} Moran^{\prime }sI=\frac{\overset{n}{\underset{i=1}{\sum }}\overset{n}{\underset{j=1}{\sum }}W_{ij}\left(X_{i}-\bar{X}\right)\left(X_{j}-\bar{X}\right)}{\overset{n}{\underset{i=1}{\sum }}\overset{n}{\underset{j=1}{\sum }}W_{ij}\overset{n}{\underset{i=1}{\sum }}{\left(X_{i}-\bar{X}\right)}^{2}} \end{array}$$

(10) $$\begin{array}{l} \;=\frac{\overset{n}{\underset{i=1}{\sum }}\overset{n}{\underset{j=1}{\sum }}w_{ij}\left(X_{i}-\bar{X}\right)\left(X_{j}-\bar{X}\right)}{s^{2}\overset{n}{\underset{i=1}{\sum }}\overset{n}{\underset{j=1}{\sum }}W_{ij}} \end{array}$$

*s*^2^ is the variance of *X*. $\bar{X}$ is the square root of *X*, n is the total number of space units, *W*_*ij*_ is the element of space weight matrix,*X*_*i*_ is the observed value of space unit *i*.

### Variable selection and data sources

#### Enterprise productivity

This study uses the China Industrial Enterprise Database. It covers more than 90% of China's enterprises. China Industrial Enterprise Database is China's most authoritative database for measuring enterprise productivity. As the latest statistical year of the China industrial enterprise database is 2014, the database of Chinese industrial enterprises from 2005 to 2014 is used to calculate enterprise productivity. Data is processed by the common processing methods (27). Oley-Pakes's semi-parametric estimation method is used to estimate enterprise productivity, and Levinsohn-Petrin's semi-parametric estimation method tests the robustness. After the TFP of each enterprise is estimated, the weighted average is made according to the proportion of the enterprise's sales in the city. The weighted average TFP of each city is obtained (28). China industrial Enterprise database needs to match with the China Patent database to measure enterprise innovation quality's mediating variable. So, in the end, ~230,000 enterprises participated in the measurement of urban enterprise productivity.

#### Air quality improvement

Air quality improvement is a composite indicator based on various emissions (29). First, we calculate the emission intensity of air pollution in each city. $I_{ijt}=\frac{P_{ijt}}{Y_{it}}/\frac{1}{n}\sum_{1}^{n}\frac{P_{ijt}}{Y_{it}}$, *I*_*ijt*_ is the emission intensity of type j pollutant in the city I and period T. *P*_*ijt*_ is the emission of type *j* pollutant in the city *I* and period *T*. *Y*_*it*_ is the total industrial output of city *I* in period *T*. Second, the intensity of air pollution emissions is averaged, $I_{it}=\frac{1}{m}\overset{m}{\underset{j=1}{\sum }}I_{ijt}$. This study uses urban industrial emissions of sulfur dioxide, industrial dust and emissions to measure urban air pollution. Finally, the comprehensive index of air quality improvement is calculated by three kinds of air pollution emissions, $AQI_{it}=\frac{1}{I_{it}}$. The higher the value is, the better the improvement effect of air quality is. The logarithm of air quality improvement was taken to alleviate heteroscedasticity.

#### Mediated variable

The first mediated variable is enterprise innovation quality (IQ). First, China Industrial Enterprise and China Patent databases are processed based on Brandt's method (27). The two databases are then matched based on the unique enterprise identifier and year for each enterprise's total number of patents. Approximately 230,000 corporate data were retained through matching; finally, according to the algorithm of urban enterprise productivity, the weighted average enterprise innovation quality is obtained. The second mediated variable is human capital (HC). Existing literature believes that employees without higher education are usually not in the scope of human capital statistics (30). Therefore, this study measures human capital by the proportion of higher education to the labor force. The third mediated variable, population health (PH), is mainly measured by mortality, life expectancy, and the incidence of certain diseases in the existing literature. Since these data are only collected at the provincial level, it is difficult to accurately and effectively measure the health level of urban residents. In this study, the proportion of urban medical expenditure per GDP is used to measure the health level of urban residents. The ratio of the city per medical expenditure to per GDP is used to measure the health level of urban residents.

#### Control variables

Based on Shiyi and Dengke (31), select Growth Rate of Population (PGR): Births minus deaths and then divided by the total population. Health level (HL): beds for every thousand citizens. The degree of opening-up (FTD): total import and export trade accounted for GDP. Number of technical employees (TE): number of persons employed in technical services and geological survey. These control variables are used to reduce further the bias of research results caused by omitted variables. Descriptive statistics of each variable are shown in Table 1. The minimum productivity of Urban enterprises in China is 4.865, and the maximum productivity is 10.750. There are certain differences among cities. The minimum value of air quality improvement is 2.126, and the maximum value is 9.638. There are also great differences in air quality improvement among different cities. The data in this study are mainly from the China Statistical Yearbook, China Urban Statistical Yearbook, China Industrial Enterprise Database, China Patent Database, official websites of provincial and municipal statistics bureau, National Research Website, and EPS website. The mean replaces the missing values.

**Table 1:** Statistical description of variables.

**Variable name**	**Mean**	**Std. Dev**.	**Median**	**Min**	**Max**
TFP	8.213	0.985	8.272	4.865	10.750
lnAQI	6.027	0.939	5.975	2.126	9.638
IQ	14.350	96.770	1.152	0.001	1943
HC	13.640	12.260	9.944	0.282	85.390
PH	28.520	16.160	25.86	1.669	116.900
PG (%)	5.874	4.923	5.385	−8.900	40.780
HL	3.574	1.581	3.326	0.814	13.580
FTD	0.221	0.435	0.089	0.001	8.134
TE	0.849	2.170	0.340	0.001	81.000

This table reports the variable's symbol, mean, standard deviation (Std. Dev.), minimum (Min.), maximum (Max.), median.

## Empirical results and analysis

### Dynamic analysis of enterprise productivity

The Gaussian Kernel density function is analyzed to further characterize the dynamic evolution trend of urban enterprise productivity in China and the eastern, central, and western cities. As shown in Formula (12), *f*(*x*) is the density function of labor productivity,


(11) $$\begin{array}{l} f\left(x\right)=\frac{1}{Nh}\overset{N}{\underset{i=1}{\sum }}K\left(\frac{X_{i}-x}{h}\right) \end{array}$$

**Figure 1:**
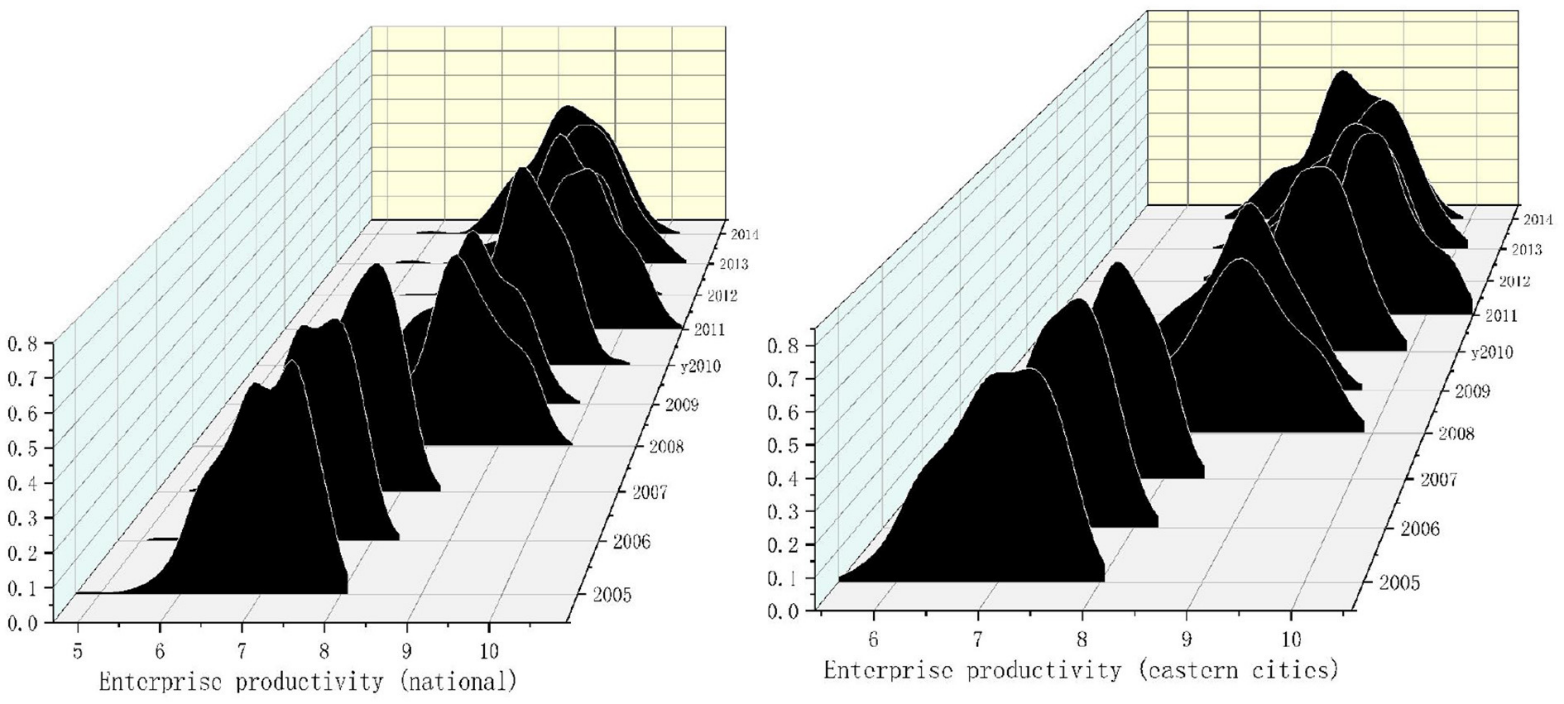
Kernel density of enterprise productivity in national and eastern cities from 2005 to 2014. Note: the closer the kernel density graph to the right, the higher the productivity. The higher the kernel density graph, the more concentrated the productivity.

**Figure 2:**
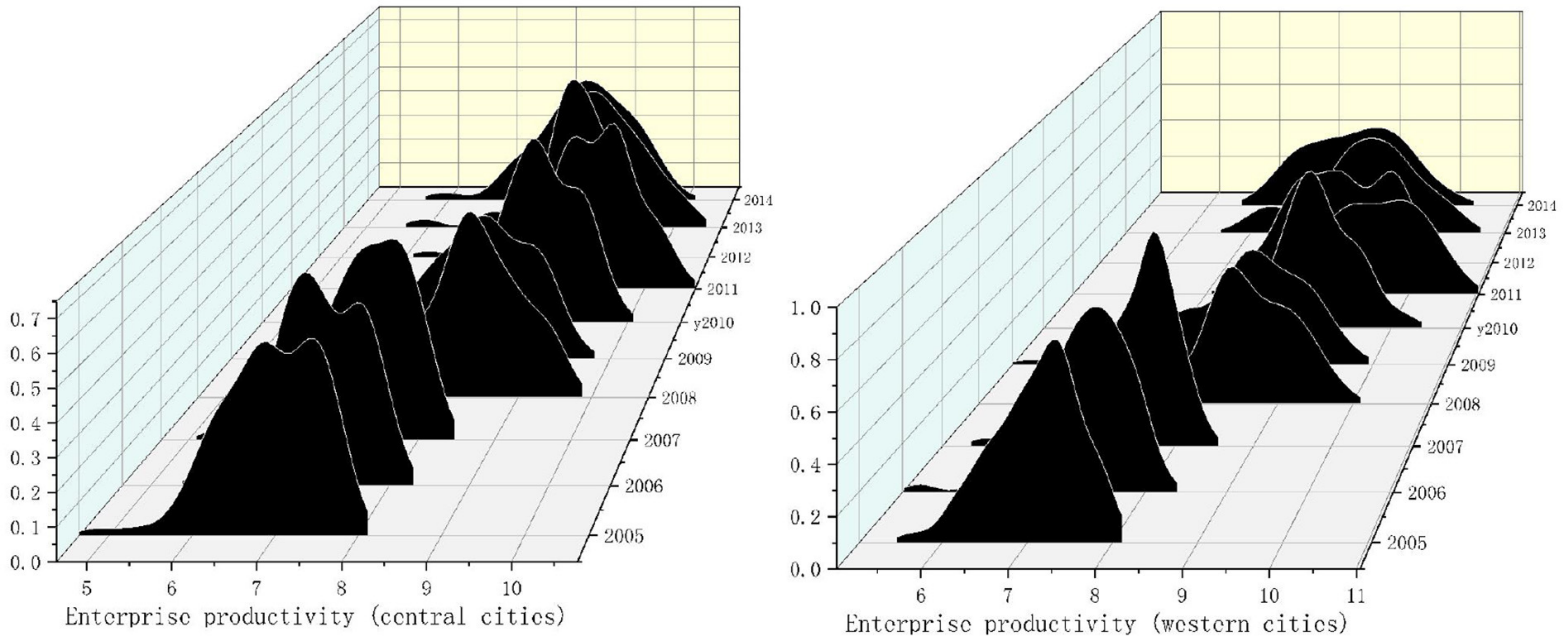
Kernel density of enterprise productivity in central and western cities from 2005 to 2014. Note: the closer the kernel density graph to the right, the higher the productivity. The higher the kernel density graph, the more concentrated the productivity.

*k*(·) is the h-dimensional kernel; *k*(·) is the product of one-dimensional kernels. *N* is the number of observed values. *X* is the mean of the observed value, *h* is the optimal bandwidth. The smaller the bandwidth, the higher the estimation accuracy. Figures 1, 2, respectively, show the dynamic changes in enterprise productivity from 2005 to 2014.

From Figure 1, the productivity of enterprises in Chinese national cities show a trend of “right-left-right.” This shows that enterprise productivity presents a fluctuating state of the first rise, then decline, and then rise. From 2005 to 2014, there was only one main peak and no flattening trend. This shows no polarization trend in urban enterprise productivity, and the productivity difference between cities is further narrowing.

The productivity of enterprises in eastern cities also shows a trend of “right-left-right.” It shows that the productivity of urban enterprises in eastern cities rises first, then declines, and then rises. Numerically, urban enterprises' productivity in eastern cities is slightly higher than the national average. There is only one main peak in the productivity density map of enterprises in the eastern cities. It shows no polarization in the productivity of urban enterprises in eastern cities.

As can be seen from Figure 2, the main peak of enterprise productivity distribution in central cities presents a “right-left-right” distribution. It shows that the productivity of enterprises in central cities increases first, then decreases, and then increases. There were two main peaks in 2005, 2006, and 2011, and the phenomenon disappeared after 2011, showing a single main peak. The results show two kinds of differentiation in the middle part of China, and the latter polarization disappeared, then the regional differences decreased. The main peak in western cities moved to the right, indicating that the productivity of urban enterprises in western cities is rising. In 2012, there was a double main peak phenomenon, and the other years were a single main peak phenomenon. It shows two kinds of polarization in western China in some years, and the polarization disappears in the later period. It shows no polarization in the productivity of urban enterprises in western cities. However, the distribution chart shows a flattening trend, indicating certain differences in enterprise productivity among cities in western China.

### Spatial correlation analysis of air quality improvement

This section mainly uses Moran's I to test the spatial correlation of air quality improvement. The specific measurement results are shown in Table 2. All the improvement coefficients of air quality have passed the significance test, indicating that air quality improvement has a significant spatial effect.

**Table 2:** Air quality improvement Moran index.

**Year**	**Air quality improvement**		
	**I**	**z**	*p*-value^*^
2005	0.259	8.340	0.000
2006	0.269	8.655	0.000
2007	0.299	9.596	0.000
2008	0.301	9.653	0.000
2009	0.303	9.714	0.000
2010	0.243	7.837	0.000
2011	0.181	5.882	0.000
2012	0.182	5.893	0.000
2013	0.157	5.111	0.000
2014	0.142	4.632	0.000

This table reports the Moran's I estimation results;

* , **, *** denote the significance at 10, 5, 1% levels, respectively.

**Figure 3:**
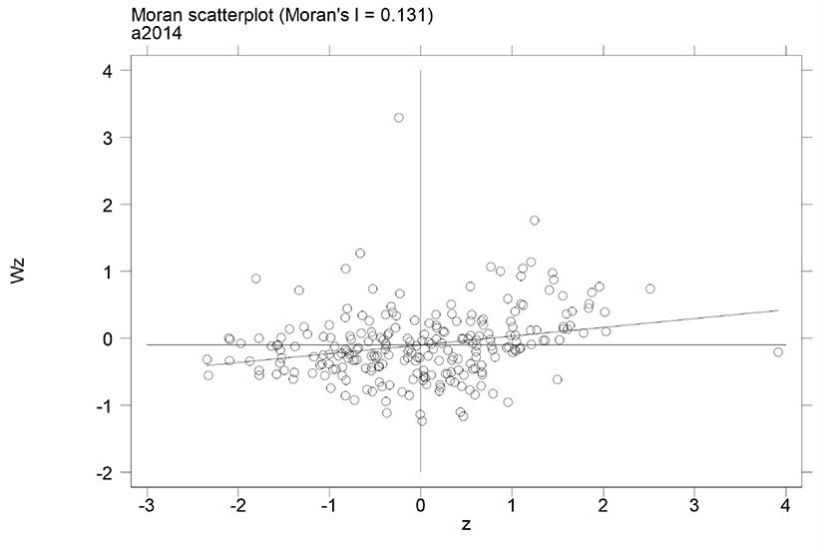
Scatter plot of the Moran index for air quality improvement, If scattered points are distributed in each quadrant, it indicates that air quality improvement has significant spatial correlation.

The Moran's I scatter plot of air quality improvement was drawn to observe the spatial accumulation characteristics of air quality improvement. From Figure 3, Moran's I of air quality improvement is distributed in each quadrant. This shows that the spatial correlation of air quality improvement is strong. The improvement of air quality in most cities shows a spatial correlation of high-high, low-low, high-low, and low-high. There are significant spatial dependence and high spatial agglomeration characteristics of air quality improvement.

### Analysis of model results

The test results of the Spatial Durbin Model for air quality improvement are shown in Table 3. The Column (1) shows regression results with no spatial effect, and the Column (2) shows regression results with spatial effect. The spatial coefficient of the model ρ is significant, and the spatial coefficient of air quality improvement is significant.

**Table 3:** SDM model regression estimation results.

**Variables**	**(1)**	**(2)**	**(3)**
	**Main**	**Wx**	**OLS**
lnAQI	0.240^***^ (14.020)	1.235^***^ (3.913)	0.142^***^ (2.802)
PGR	0.013^***^ (4.442)	0.051 (1.395)	−0.020^***^ (−4.098)
HL	0.086^***^ (8.229)	0.684^***^ (3.154)	0.586^***^ (28.130)
FTD	−0.072^**^ (−2.094)	3.540^***^ (4.147)	0.014 (0.192)
TE	0.003 (0.585)	−0.041 (−0.149)	0.009 (0.612)
ρ	−1.055^***^ (−2.789)		
Obs	2,420	2,420	2,420
R-squared	0.257	0.257	0.275
Number of city	242	242	242

This table reports the estimation results of SDM model. T-stat values are reported in parentheses ();

*, **, *** denote the significance at 10, 5, 1% levels, respectively.

Since ρ is not equal to zero in the model, coefficients in the model cannot directly explain the economic significance of variables. Therefore, it is necessary to decompose the effect of air quality improvement. It can be divided into direct, indirect, and total effects. The decomposition is shown in Table 4.

**Table 4:** Decomposition effect.

**Variable**	**Direct effect**	**Indirect effect**	**Total effect**
lnAQI	0.237^***^ (14.150)	0.519^**^ (2.563)	0.756^***^ (3.675)
PGR	0.013^***^ (5.121)	0.021 (0.978)	0.033 (1.514)
HL	0.084^***^ (7.967)	0.304^**^ (2.335)	0.388^***^ (2.955)
FTD	−0.081^**^ (−2.117)	1.859^***^ (2.744)	1.778^***^ (2.586)
TE	0.002 (0.344)	−0.021 (−0.139)	−0.018 (−0.123)

This table reports the results of decomposition effect. T-stat values are reported in parentheses ();

*, **, *** denote the significance at 10, 5, 1% levels, respectively.

#### Direct effect

Table 4 shows that the direct effect of air quality improvement is 0.237, which is significant at the 1% level. The results show that local areas' air quality improvement can significantly improve local areas' enterprises' productivity increase. Conversely, the improvement of air quality requires enterprises to reduce emissions of polluting gases, forcing them to improve energy efficiency and production technology, reduce undesirable output, and then promote the improvement of enterprise productivity. In contrast, the improvement of air quality makes the urban air environment more livable, ensures the health of residents, promotes the formation of urban health human capital, and then promotes enterprises' productivity increase.

#### Indirect effect

Table 4 shows that the indirect effect of air quality improvement is 0.519, which is significant at a 5% level. The results show that the improvement of adjacent areas' air quality significantly promotes local areas' enterprises' productivity. Conversely, the improvement of air quality in adjacent areas leads to lower air pollutants emissions, thus reducing the number of pollutants scattered in the local area. So the degree of air pollution suffered by residents and enterprises in the local area has been reduced. Thus, this promotes the productivity of enterprises in the local area. In contrast, adjacent areas' air quality improvement forces enterprises to improve their production technology. The advanced production technology has certain positive externalities. Therefore, when the advanced production technology in the adjacent areas spills over to the local area, it promotes the upgrading of production technology in the local area, thus promoting local areas' enterprise productivity increase.

#### Total effect

The total effect is the sum of direct and indirect effects. Table 4 shows that the total effect of air quality improvement on enterprise productivity is 0.756, which is significant at the 1% level. The results show that the improvement of air quality can significantly improve enterprise productivity. From the third column of Table 3, the OLS result shows that the impact of air quality improvement on enterprise productivity is significantly positive, and the coefficient is 0.142. However, the coefficient is 0.756 when the spatial spillover effect is considered. The results showed that OLS underestimated the effect of air quality improvement on enterprise productivity when it ignored the spatial spillover effect.

The direct effect of the control variable PGR is significantly positive. This indicates that the population growth in this region provides sufficient labor for enterprise development and promotes local areas' enterprise productivity increase. The total effect of HL is significantly positive, indicating that HL can promote enterprise productivity increase. Improved HL has ensured the health of residents and allowed the stock of human capital to increase further, boosting the productivity of enterprises. The total effect of the control variable FTD is significantly positive, indicating that FTD can promote the improvement of enterprise productivity. Local enterprises are forced to introduce advanced technology, reduce enterprise costs, improve the competitiveness of their products, and thus promote the improvement of enterprise productivity owing to the influx of foreign products into the local market. TE did not pass the significance test, possibly because its promoting effect was not obvious in this study.

### Heterogeneity analysis

This section divides the sample into the eastern, central, and western regions. The periods are divided into 2005–2010, 2010–2014 to investigate whether the impact of air quality improvement on enterprise productivity varies with different regions and periods.

#### Regional heterogeneity

This section divides China into eastern, central, and western regions for investigation because of the differences in economic development, climate, and customs in different regions of China. It discusses whether there are differences in the impact of air quality improvement on enterprise productivity in different regions. The results are shown in Table 5.

**Table 5:** Impact of air quality improvement on enterprise productivity in eastern, central and western China.

**Variables**	**(1)**	**(2)**	**(3)**	**(4)**	**(5)**	**(6)**
	**Eastern cities**		**Central cities**		**Western cities**	
	**Main**	**Wx**	**Main**	**Wx**	**Main**	**Wx**
lnAQI	0.170^***^ (6.190)	1.778^***^ (3.050)	0.416^***^ (11.610)	1.564^**^ (2.460)	0.312^***^ (8.090)	2.080^***^ (2.578)
Control Vars	Yes	Yes	Yes	Yes	Yes	Yes
	−0.991^***^ (−3.415)		−0.902^***^ (−3.762)		−0.590^*^ (−1.761)	
Obs	930		880		610	
R-squared	0.286		0.204		0.196	
Number of city	93		88		61	

This table reports the estimation results of different regions. T-stat values are reported in parentheses ();

*, **, *** denote the significance at 10, 5, 1 levels, respectively.

Table 5 shows that ρ has passed the 10% significance test, which indicates that the air quality improvement of cities in eastern, central, and western regions has a significant spatial effect. The decomposition effect is shown in Table 6.

**Table 6:** Decomposition effect.

**Variables**	**(1)**	**(2)**	**(3)**	**(4)**	**(5)**	**(6)**	**(7)**	**(8)**	**(9)**
	**Eastern cities**			**Central cities**			**Western cities**		
	**Direct effect**	**Indirect effect**	**Total effect**	**Direct effect**	**Indirect effect**	**Total effect**	**Direct effect**	**Indirect effect**	**Total effect**
lnAQI	0.155^***^	0.874^**^	1.029^***^	0.404^***^	0.690^*^	1.094^***^	0.298^***^	1.394^*^	1.692^**^
Control Vars	Yes	Yes	Yes	Yes	Yes	Yes	Yes	Yes	Yes

This table reports the results of decomposition effect. T-stat values are reported in parentheses ();

*, **, *** denote the significance at 10, 5, 1% levels, respectively.

Table 6 shows that the direct, indirect, and total effects of air quality improvement in eastern, central, and western regions all pass the significance test. The results show that the improvement of air quality has a significant direct effect and a spatial spillover effect in eastern, central, and western cities. In terms of the total effect, the western cities are significantly higher than the eastern and central cities. Enterprise development is extensive owing to the low economic development and industrial structure in western cities. Therefore, the improvement of air quality in western cities has a more obvious effect on enterprise productivity.

#### Time heterogeneity

In 2010, China promulgated compiling and publishing technical guidelines on environmental protection standards, technical guidelines for the standard formulation, and revision of environmental monitoring. These environmental regulations mark China's more detailed and stringent approach to air pollution control. Therefore, this section takes 2010 as the time segmentation point to investigate whether the impact of air quality improvement on enterprise productivity is different because of the intensity of air pollution prevention and control. The results are shown in Table 7.

**Table 7:** Impact of air quality improvement on enterprise productivity at 2005–2010 and 2010–2014.

**Variables**	**(1)**	**(2)**	**(3)**	**(4)**
	**2005–2010**		**2010–2014**	
	**Main**	**Wx**	**Main**	**Wx**
*lnAQI*	0.236^***^ (11.02)	1.915^***^ (3.166)	0.203^***^ (6.030)	1.013^***^ (3.165)
Control Vars	Yes	Yes	Yes	Yes
ρ	−0.923^*^ (−1.941)		0.820^***^ (12.740)	
Observations	1,452	1,452	1,210	1,210
R-squared	0.261	0.261	0.293	0.293
Number of id	242	242	242	242

This table reports the estimation results of different times. T-stat values are reported in parentheses ();

*, **, *** denote the significance at 10, 5, 1% levels, respectively.

Table 7 shows that air quality improvement still passes the significance test in different years, indicating that air quality improvement still has a significant spatial impact on enterprise productivity in different years. The decomposition effects are further analyzed in Table 8.

**Table 8:** Decomposition effect.

**Vairable**	**(1)**	**(2)**	**(3)**	**(4)**	**(5)**	**(6)**
	**2005–2010**			**2010–2014**		
	**Direct effect**	**Indirect effect**	**Total effect**	**Direct effect**	**Indirect effect**	**Total effect**
lnAQI	0.231^***^ (11.080)	0.990^**^ (2.219)	1.222^***^ (2.721)	0.229^***^ (5.748)	7.283^**^ (2.038)	7.512^**^ (2.088)
Control Vars	Yes	Yes	Yes	Yes	Yes	Yes

This table reports the results of decomposition effect. T-stat values are reported in parentheses ();

*, **, *** denote the significance at 10, 5, 1% levels, respectively.

Table 8 shows that the total effect of air quality improvement on enterprise productivity has passed the significance test. It shows that the spatial spillover effect of air quality improvement still exists in different periods. However, from the perspective of coefficient size, the total coefficient effect of air quality improvement in 2010–2014 is much higher than in 2005–2010. It shows that the improvement of air quality has a more obvious promoting effect on enterprise productivity when the intensity of air pollution control increases.

### Endogeneity test

The space instrumental variable method is difficult to solve the model endogeneity problem because of its complexity and weight matrix endogeneity problem. This is because of the endogeneity problem in the air quality improvement model. Therefore, in addition to controlling some urban characteristic variables, the instrumental variable method is used to alleviate the endogeneity problems existing in the model. Considering that air pollution control has significant spatial spillover effects and that air has the nature of diffusion, referring to the research of Broner et al. (32) and Hering and Poncet (33), the air circulation coefficient is chosen as the first instrumental variable of air quality improvement. Environmental regulation does not directly affect enterprise productivity, but the intensity of environmental regulation will directly affect the improvement degree of air quality. Therefore, environmental regulation is chosen as the second instrumental variable (31). For the measurement of environmental regulation, the existing literature mostly uses environmental input, sewage cost, and several environmental protection employees (34, 35). It is difficult to reflect on the idea of governance, and to a certain extent, the selection of these indicators is inherent in improving enterprise productivity. The externality of instrumental variables is difficult to satisfy. Therefore, the frequency of environmental protection words in municipal government reports is used to measure environmental regulations (36, 37). The correlation between environmental regulation and air quality improvement needs no elaboration. The main reason why environmental regulations meet externality is that government reports are generally released at the beginning of the year, while enterprises' production activities are carried out throughout the whole year. It can effectively avoid endogeneity problems caused by “reverse causality.” The equation of the air circulation coefficient is as follows:


(12) $$\begin{array}{l} VC=WS\times BLH \end{array}$$

**Figure 4:**
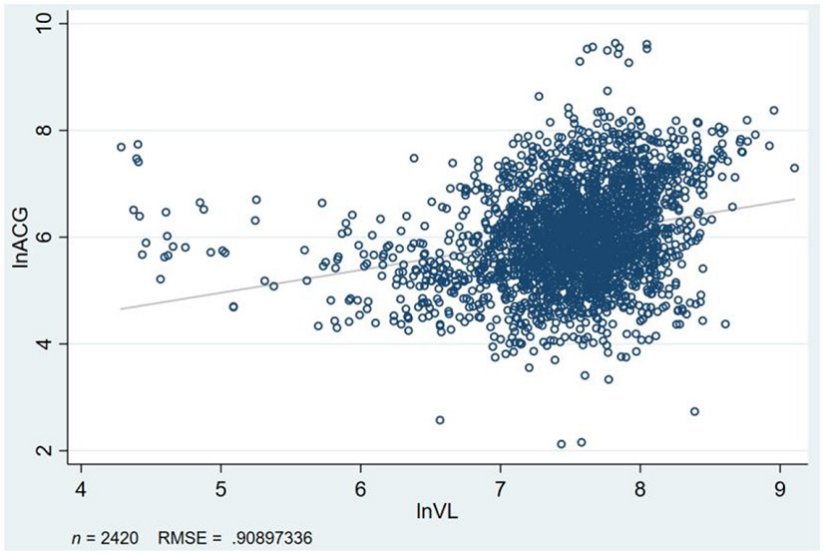
Air quality improvement and air flow coefficient scatter diagram. Figure shows that the two variables have the same tendency.

Where VC is air circulation coefficient, WS is wind speed, and BLH is atmospheric boundary height. Figure 4 shows a strong positive correlation between airflow coefficient and atmospheric quality improvement, which meets the correlation premise of instrumental variables. At the same time, airflow coefficient is mainly affected by geographical location, such as atmospheric height and meteorological system. The externality premise of instrumental variables is well-satisfied; the results are shown in Table 9.

**Table 9:** Instrumental variable regression result.

	**(1)**	**(2)**
	**First stage regression results**	**Second stage regression results**
Vairable	lnAQI	TFP
l.lnVL	0.382^***^ (0.118)	
ER	0.005^**^ (0.002)	
lnAQI		0.247^*^ (0.145)
Control Vars	Yes	Yes
Obs	3,406	3,406
Ctiy	262	262
Under-identification test		*P* = 0.001
Weak identification test		*F* = 79.433
Over-identification test		*P* = 0.594

This table reports the estimation results of endogeneity test and robustness test. In column (1) we present the results of the first-stage regression, In column (2), we show the second-stage regression results and the effectiveness of instrumental variable selection, robust standard errors in parentheses ();

*, **, *** denote the significance at 10, 5, 1% levels, respectively.

From Table 9, the regression results of the first stage show that the two instrumental variables are highly correlated with air quality improvement. The instrumental variables have passed the under-identification test, the weak identification test, and the over-identification test, indicating that the two instrumental variables are valid. According to the results, the coefficient of air quality improvement in the instrumental variable method is 0.168, lower than the regression coefficient of 0.756 in the Spatial Dubin Model. Owing to the endogeneity of the Spatial Dubin Model, it overestimates the impact of air quality improvement on enterprise productivity.

### Robustness test

In this section, robustness tests are carried out by replacing core explanatory variables, changing spatial weight matrices, and using different spatial models.

#### Replace core explanatory variables

The text uses Oley-Pakes semi-parameter estimation method to estimate enterprise productivity. Here, the Levinsohn-Petrin semi-parameter is used to estimate the enterprise productivity of each city. The results are shown in Table 10.

**Table 10:** Impact of atmospheric quality improvement on enterprise productivity—LP.

**Vairable**	**(1)**	**(2)**	**(3)**	**(4)**	**(5)**
	**Main**	**Wx**	**Direct effect**	**Indirect effect**	**Total effect**
lnAQI	0.264^***^ (14.09)	1.313^***^ (3.781)	0.261^***^ (14.220)	0.530^**^ (2.543)	0.791^***^ (3.732)
Control Vars	Yes (1.353)	Yes (−0.032)	Yes (0.991)	Yes (−0.044)	Yes (0.003)
ρ	−1.095^***^ (−2.925)				
Obs	2,420	2,420	2,420	2,420	2,420
R-squared	0.304	0.304	0.304	0.304	0.304
Number of id	242	242	242	242	242

This table reports the estimation results of SDM model and decomposition effect. T-stat values are reported in parentheses ();

*, **, *** denote the significance at 10, 5, 1% levels, respectively.

Table 10 shows that direct, indirect, and total effects have no significant changes in significance and coefficient direction, indicating that this study's conclusion is robust and reliable.

#### Replace the weight matrix

The text mainly uses a spatial adjacent weight matrix for regression. This section uses the economic distance weight matrix for the robustness test. The results are shown in Table 11. It can be seen that the coefficient direction and significance of direct, indirect, and total effects do not change. It can be concluded that the research conclusion has certain robustness.

**Table 11:** Impact of air quality improvement on enterprise productivity—weight matrix of economic distance.

**Vairable**	**(1)**	**(2)**	**(3)**	**(4)**	**(5)**
	**Main**	**Wx**	**Direct effect**	**Indirect effect**	**Total effect**
lnAQI	0.099^***^ (5.878)	0.237^***^ (5.585)	0.099^***^ (5.685)	0.227^***^ (5.902)	0.327^***^ (8.064)
Control Vars	Yes	Yes	Yes	Yes	Yes
ρ	−0.030 (−0.732)				
Obs	2,420	2,420	2,420	2,420	2,420
R-squared	0.161	0.161	0.161	0.161	0.161
Number of id	242	242	242	242	242

This table reports the estimation results of SDM model and decomposition effect. T-stat values are reported in parentheses ();

*, **, *** denote the significance at 10, 5, 1% levels, respectively.

#### Alternate regression method

This study mainly uses SDM to test the spatial spillover effect of air quality improvement on enterprise productivity. SAR and SEM models are mainly used for robustness tests in this section to test their spatial effect. The results are shown in Table 12.

**Table 12:** SAR and SEM model regression results of air quality improvement.

**Variable**	**(1)**	**(2)**	**(3)**	**(4)**	**(5)**
	**SAR**				**SEM**
	**Main**	**Direct effect**	**Indirect effect**	**Total effect**	**Main**
lnAQI	0.132^***^ (5.851)	0.140^***^ (5.884)	1.959^***^ (4.212)	2.099^***^ (4.364)	0.200^***^ (12.370)
Control Vars	Yes	Yes	Yes	Yes	Yes
ρ	0.936^***^ (83.07)				−2.551^***^ (−15.10)
Obs	2,420	2,420	2,420	2,420	
R-squared	0.303	0.303	0.303	0.303	
Number of city	242	242	242	242	

This table reports the estimation results of SAR, SEM model and decomposition effect of SAR. T-stat values are reported in parentheses ();

*, **, *** denote the significance at 10, 5, 1% levels, respectively.

Table 12 shows that the spatial effect of air quality improvement on enterprise productivity still exists significantly in SAR and SEM models. There is no significant change in the coefficient and significance in the SAR model after effect decomposition. Therefore, the research conclusion of this study is robust and reliable.

### Additional analysis

To further explore the channel effect of air quality improvement on enterprise productivity, this section mainly discusses the three channels; enterprise innovation quality, human capital, and residents' health.

#### Enterprise innovation quality

Formulas (13) and (14) are added to the basic regression formula (6) to represent the mediating effect of enterprise innovation quality. The specific formula is as follows:


(13) $$\begin{array}{l} TFP_{it}=\alpha_{0}+\delta W\cdot TFP_{it}+\alpha_{1}\ln\;AQI_{it}+\alpha_{2}W\cdot \ln\;AQI_{it}\\ \;+\alpha_{3}IQ_{it}+\alpha_{4}W\cdot IQ_{it}\\ \;+\alpha_{5}W\cdot X_{control}+\alpha_{6}X_{control}+\mu_{it} \end{array}$$

(14) $$\begin{array}{l} \begin{array}{l} IQ_{it}=\varphi_{0}+\delta W\cdot IQ_{it}+\varphi_{1}\ln\;AQI_{it}+\varphi_{2}W\cdot \ln\;AQI_{it} \end{array}\\ \;\begin{array}{l} +\varphi_{3}W\cdot X_{control}+\varphi_{4}X_{control}+\mu_{it} \end{array} \end{array}$$

The model results are shown in Table 13. Columns (1) and (2) are the basic regressions of this study, reflecting the impact of air quality improvement on enterprise productivity. Columns (5) and (6) reflect the impact of air quality improvement on enterprise innovation quality and the regression result of the formula (14). Columns (3) and (4) are the regression results of formula (13), indicating that enterprise innovation and air qualities improvement are put into the model at the same time. This is to investigate whether the mediating effect of enterprise innovation quality exists. Table 13 shows that the spatial coefficients are significant, so the model has a significant spatial effect. Specific effect decomposition results are shown in Table 14.

**Table 13:** Thet estimation results of mediating variable—enterprise innovation quality.

**Vairable**	**(1)**	**(2)**	**(3)**	**(4)**	**(5)**	**(6)**
	**TFP**		**TFP**		**IQ**	
	**Main**	**Wx**	**Main**	**Wx**	**Main**	**Wx**
IQ			0.001^***^ (2.618)	0.018^**^ (2.548)		
lnAQI	0.240^***^ (14.020)	1.235^***^ (3.913)	0.157^***^ (6.828)	0.300 (1.340)	10.37^***^ (4.999)	87.48^**^ (2.301)
Control Vars	Yes	Yes	Yes	Yes	Yes	Yes
ρ	−1.055^***^ (−2.789)		0.919^***^ (58.30)		−0.681^*^ (−1.740)	
Obs	2,420		2,420		2,420	
R-squared	0.257		0.388		0.166	
Number of city	242		242		242	

This table reports the estimation results of enterprise innovation quality which is the mediating variable of air quality improvement on enterprise productivity. T-stat values are reported in parentheses ();

*, **, *** denote the significance at 10, 5, 1% levels, respectively.

**Table 14:** Decomposition effect of enterprise innovation quality.

**Vairable**	**(1)**	**(2)**	**(3)**	**(4)**	**(5)**	**(6)**	**(7)**	**(8)**	**(9)**
	**TFP**			**TFP**			**IQ**		
	**Direct effect**	**Indirect effect**	**Total effect**	**Direct effect**	**Indirect effect**	**Total effect**	**Direct effect**	**Indirect effect**	**Total effect**
IQ				0.002^***^ (3.389)	0.232^**^ (2.553)	0.234^**^ (2.559)			
lnAQI	0.237^***^ (14.150)	0.519^**^ (2.563)	0.756^***^ (3.675)	0.179^***^ (6.267)	5.915^*^ (1.650)	6.094^*^ (1.691)	10.270^***^ (4.882)	51.540^*^ (1.825)	61.810^**^ (2.133)
Control Vars	Yes	Yes	Yes	Yes	Yes	Yes	Yes	Yes	Yes

This table reports the decomposition effect of enterprise innovation quality. T-stat values are reported in parentheses ();

*, **, *** denote the significance at 10, 5, 1% levels, respectively.

Columns (1), (2), and (3) in Table 14 are the results of basic regression. Columns (7), (8), and (9) reflect the impact of air quality improvement on enterprise innovation quality. Column (7) reflects that the direct effect coefficient is significantly positive, indicating that the improvement of air quality can significantly improve enterprise innovation quality in the local area. The improvement of air quality forces local enterprises to upgrade their production technology, strengthen their green innovation capability and promote the improvement of their innovation quality. Column (8) reflects that the indirect effect is significantly positive, indicating that the improvement of atmospheric quality in adjacent areas promotes the improvement of enterprise innovation quality in the local area. The improvement of air quality in the adjacent area improves the technological level of enterprises in the adjacent. The positive externalities of advanced technology can be spilled to the local area. This improves the innovation quality of enterprises in the local area. Column (9) reflects the total effect of air quality improvement on enterprise innovation quality, and the total effect is significantly positive. It shows that the improvement of air quality promotes the improvement of enterprise innovation quality.

Columns (4), (5), and (6) focus on adding air quality improvement and enterprise innovation quality into the model at the same time. This is to test whether the mediating effect of enterprise innovation quality exists. Column (6) that the innovation quality coefficient of enterprises is significantly positive. It shows that the mediating effect of enterprise innovation quality exists. The improvement of air quality promotes the improvement of enterprise productivity increase by promoting the improvement of enterprise innovation quality. Meanwhile, the air quality improvement coefficient in column (6) is still significant, indicating an incomplete mediation effect. The improvement of air quality promotes the improvement of enterprise productivity. However, at the same time, air quality improvement promotes enterprise productivity by promoting enterprise innovation quality.

#### Human capital

Based on formula (6), formulas (15) and (16) are added to represent the mediating effect of human capital. The formula is as follows:


(15) $$\begin{array}{l} TFP_{it}=\gamma_{0}+\delta W\cdot TFP_{it}+\gamma_{1}\ln\;AQI_{it}+\gamma_{2}W\cdot \ln\;AQI_{it}\\ \;+\gamma_{3}HC_{it}+\gamma_{4}W\cdot HC_{it}+\\ \;\gamma_{5}W\cdot X_{control}+\gamma_{6}X_{control}+\mu_{it} \end{array}$$

(16) $$\begin{array}{l} \begin{array}{l} HC_{it}=\phi_{0}+\phi W\cdot HC_{it}+\phi_{1}\ln\;AQI_{it}+\phi_{2}W\cdot \ln\;AQI_{it} \end{array}\\ \;\begin{array}{l} +\phi_{3}W\cdot X_{control}+\phi_{4}X_{control}+\mu_{it} \end{array} \end{array}$$

The regression results of the model are shown in Table 15. Columns (1) and (2) represent the regression results of the basic model. Columns (5) and (6) reflect the impact of air quality improvement on human capital and the regression results of formula (16). Columns (3) and (4) tests are to ensure air quality improvement and human capital in the model at the same time to verify whether the mediation effect of human capital exists, which is also the regression result of formula (15). Table 15 shows that the spatial coefficient of the model is significantly not 0, indicating that the spatial effect exists. The specific decomposition effect is shown in Table 16.

**Table 15:** The estimation results of mediating variable—human capital.

**Vairable**	**(1)**	**(2)**	**(3)**	**(4)**	**(5)**	**(6)**
	**TFP**		**TFP**		**HC**	
	**Main**	**Wx**	**Main**	**Wx**	**Main**	**Wx**
HC			0.002 (1.030)	0.058^***^(4.110)		
lnAQI	0.240^***^ (14.020)	1.235^***^ (3.913)	0.174^***^ (7.475)	1.040^***^ (4.092)	1.772^***^ (5.537)	2.572 (0.439)
Control Vars	Yes	Yes	Yes	Yes	Yes	Yes
ρ	−1.055^***^ (−2.789)		0.861^***^ (33.290)		−0.936^**^ (−2.283)	
Obs	2,420		2,420		2,420	
R-squared	0.257		0.566		0.057	
Number of city	242		242		242	

This table reports the estimation results of human capital which is the mediating variable of air quality improvement on enterprise productivity. T-stat values are reported in parentheses ();

*, **, *** denote the significance at 10, 5, 1% levels, respectively.

**Table 16:** Decomposition effect of human capital.

**Vairable**	**(1)**	**(2)**	**(3)**	**(4)**	**(5)**	**(6)**	**(7)**	**(8)**	**(9)**
	**TFP**			**TFP**			**HC**		
	**Direct effect**	**Indirect effect**	**Total effect**	**Direct effect**	**Indirect effect**	**Total effect**	**Direct effect**	**Indirect effect**	**Total effect**
HC				0.003^*^ (1.910)	0.429^***^ (6.506)	0.433^***^ (6.543)			
lnAQI	0.237^***^ (14.150)	0.519^**^ (2.563)	0.756^***^ (3.675)	0.205^***^ (8.018)	8.774^***^ (3.869)	8.979^***^ (3.936)	1.780^***^ (5.516)	0.730 (0.229)	2.511 (0.759)
Control Vars	Yes	Yes	Yes	Yes	Yes	Yes	Yes	Yes	Yes

This table reports the decomposition effect of human capital. T-stat values are reported in parentheses ();

*, **, *** denote the significance at 10, 5, 1% levels, respectively.

Columns (1), (2), and (3) in Table 16 reflect the impact of the basic model. Columns (7), (8), and column (9) reflect the impact of air quality improvement on human capital. According to columns (7) and (8), it is found that the direct effect of air quality improvement on human capital is significant while the indirect effect is not. It shows that the direct effect is the main channel of air quality improvement to promote human stock capital in the local area. The amount of labor inflow determines the human stock capital of a city. Air quality improvement improves the local area's living and working environment. The adjacent area's migration intention of labor is enhanced, promoting the increase of the human stock capital of local areas. Columns (4), (5), and (6) focus on whether the mediating effect of human capital exists when air quality improvement and human capital are both included in the model. The regression results in column (6) show that human capital is significantly positive, indicating that the mediating effect of human capital exists significantly. That is to say, air quality improvement promotes enterprise productivity increase by increasing human stock capital. Column (6) shows that the air quality improvement coefficient is also significantly positive. It shows that the mediating effect is incomplete. Air quality improvement promotes enterprise productivity increase and boosts enterprise productivity by promoting human capital stock increase.

#### Resident health

Based on formula (6), formulas (17) and (18) are added to represent the mediating effect of resident health. The formula is as follows:


(17) $$\begin{array}{l} TFP_{it}=\gamma_{0}+\delta W\cdot TFP_{it}+\gamma_{1}\ln\;AQI_{it}+\gamma_{2}W\cdot \ln\;AQI_{it}\\ \;+\gamma_{3}PH_{it}+\gamma_{4}W\cdot PH_{it}+\\ \;\gamma_{5}W\cdot X_{control}+\gamma_{6}X_{control}+\mu_{it} \end{array}$$

(18) $$\begin{array}{l} \begin{array}{l} PH_{it}=\phi_{0}+\phi W\cdot PH_{it}+\phi_{1}\ln\;AQI_{it}+\phi_{2}W\cdot \ln\;AQI_{it} \end{array}\\ \;\begin{array}{l} +\phi_{3}W\cdot X_{control}+\phi_{4}X_{control}+\mu_{it} \end{array} \end{array}$$

The model results are shown in Table 17, and columns (1) and (2) represent the regression results of the basic model. Columns (5) and (6) reflect the impact of air quality improvement on resident health and the regression results of formula (18). Columns (3) and (4) ensure air quality improvement and resident health in the model. This is to verify whether the mediation effect on resident health exists. It is also the regression result of the formula (17). Table 17 shows that the spatial coefficients of the model are significantly not 0, indicating that the spatial effect exists significantly; the decomposition effects are shown in Table 18.

**Table 17:** Thet estimation results of mediating variable—resident health.

**Vairable**	**(1)**	**(2)**	**(3)**	**(4)**	**(5)**	**(6)**
	**TFP**		**TFP**		**PH**	
	**Main**	**Wx**	**Main**	**Wx**	**Main**	**Wx**
PH			−0.014^***^ (−8.607)	−0.042^**^ (−2.259)		
lnAQI	0.240^***^ (14.020)	1.235^***^ (3.913)	0.107^***^ (4.548)	0.022 (0.070)	−0.890^***^ (−3.517)	−23.97^***^ (−7.817)
Control Vars	Yes	Yes	Yes	Yes	Yes	Yes
ρ	−1.055^***^ (−2.789)		0.915^***^ (54.22)		−1.284^***^ (−8.693)	
Obs	2,420		2,420		2,420	
R-squared	0.257		0.386		0.046	
Number of city	242		242		242	

This table reports the estimation results of resident health which is the mediating variable of air quality improvement on enterprise productivity. T-stat values are reported in parentheses ();

*, **, *** denote the significance at 10, 5, 1% levels, respectively.

**Table 18:** Decomposition effect of resident health.

**Vairable**	**(1)**	**(2)**	**(3)**	**(4)**	**(5)**	**(6)**	**(7)**	**(8)**	**(9)**
	**TFP**			**TFP**			**PH**		
	**Direct effect**	**Indirect effect**	**Total effect**	**Direct effect**	**Indirect effect**	**Total effect**	**Direct effect**	**Indirect effect**	**Total effect**
PH				−0.016^***^ (−8.443)	−0.649^***^ (−3.348)	−0.665^***^ (−3.412)			
lnAQI	0.237^***^ (14.150)	0.519^**^ (2.563)	0.756^***^ (3.675)	0.113^***^ (3.700)	1.755 (0.441)	1.868 (0.467)	−0.798^***^ (−3.085)	−10.03^***^ (−9.688)	−10.83^***^ (−10.360)
Control Vars	Yes	Yes	Yes	Yes	Yes	Yes	Yes	Yes	Yes

This table reports the decomposition effect of resident health. T-stat values are reported in parentheses ();

*, **, *** denote the significance at 10, 5, 1% levels, respectively.

Columns (1), (2), and (3) in Table 18 reflect the impact of the basic model. Columns (7), (8), and (9) reflect the impact of air quality improvement on resident health. Column (7) shows that the direct effect of air quality improvement on resident health expenditure is significantly negative, indicating that air quality improvement significantly reduces residents' health expenditure. Air quality improvement reduces the incidence and hospitalization rates of asthma, respiratory and heart disease among residents, then improves the health level of the residents. The indirect benefit is also significantly negative, indicating that the adjacent area's air quality improvement reduces the medical expenditure of local areas' residents. Since air pollution has the natural property of flowing with the atmosphere, when the air quality of adjacent areas improves, the amount of air pollutants scattered into the local area also decreases. Hence, local area residents' respiratory diseases, heart disease, and death rates are reduced. At the same time, the advanced medical technology in adjacent areas will also spill over to the local area to ensure the health of residents. The total effect is negative, indicating that air quality improvement can significantly reduce the medical expenditure of residents and ensure their health. Columns (4), (5), and (6) ensure air quality improvement and resident health in the model at the same time to test whether the mediating effect of resident health exists. Column (6) shows that resident health expenditure is significantly negative, indicating that the mediating effect of resident health exists. This means that air quality improvement promotes enterprise productivity increase by improving people's health.

## Conclusions and policy implications

### Conclusions

This study is based on the combined data of China Industrial Enterprise and China Patent Databases. Firstly, the Spatial Dubin Model at the city level is innovatively used to investigate whether the spatial effect of air quality improvement on enterprise productivity improvement exists. Secondly, the impacts of air quality improvement on enterprise productivity in different regions (eastern, central, and western cities) and different periods (2005–2010 and 2010–2014) are analyzed. Finally, two instrumental variables, air flow coefficient and environmental regulation, are used to alleviate the endogeneity problem of the model. The conclusion is: (1) Air quality improvement can significantly promote enterprise productivity increase. Its spatial spillover effect can significantly promote adjacent areas' enterprise productivity increase. The spatial spillover effect of improved air quality on enterprise productivity is still significant after replacing explanatory variables, changing spatial measurement methods, and transforming the spatial matrix. (2) Heterogeneity analysis shows that the air quality improvement in western cities has a more obvious promoting effect on enterprise productivity. After 2010, the government adopted more stringent air governance measures, making air quality improvement more obvious in promoting enterprise productivity increase. Air quality improvement increases enterprise productivity by promoting the improvement of enterprises' innovation quality, ensuring the health level of urban residents, and increasing the stock of urban human capital.

### Policy implications

This study proves the importance of improving air quality from enterprise productivity. It provides the following policy enlightenment for air quality improvement.

The government should speed up the establishment of an air pollution monitoring system. The effect of air quality improvement should be included in the performance evaluation indicators of government officials. The government should strictly implement the action to prevent and control air pollution policy and strengthen oversight of enterprises with high pollution and emissions. The government actively encourages enterprises to introduce and research efficient production technologies, encouraging the use of clean and renewable energy. The “intelligent network supervision platform” is used to implement intelligent supervision of enterprises and truly implement the integration of environmental protection testing and official assessment.

(2) Implement differentiated governance measures according to different regions. Eastern cities will play a leading role in technology innovation and actively introduce advanced production technologies and innovative talents. The government gives enterprises appropriate fiscal and tax preferential policies to guide enterprises to innovate clean technology. The central and western regions should establish core cities as transfer stations for introducing advanced technology and innovative talents from the eastern cities and abroad. The government is actively implementing supporting facilities to ensure the steady increase of human capital stock.

(3) Implement a joint defense and governance system. Air pollution has a significant spatial spillover effect, so cities must implement a coordinated defense and governance system. Implement emission rights trading under prescribed emission targets, an emission compensation system, and an emission trading mechanism. A system of mutual supervision and coordinated governance will be formed among different regions to increase enterprise productivity.

This study still has certain limitations. First, only enterprise innovation quality, human capital and resident health were tested in the analysis of the influencing mechanism. In the future, appropriate influencing factors will be selected to conduct a more in-depth spatial dynamic analysis of the enterprise productivity. Second, since China Industrial Enterprise Database has only been updated to 2014, if there are updated datas in the future, we will further update the data in the following study.

## Data availability statement

Publicly available datasets were analyzed in this study. This data can be found here: The Ministry of Ecology and Environment of the People's Republic of China (https://www.mee.gov.cn/, accessed on 15 March 2022); the National Bureau of Statistics (http://www.stats.gov.cn/, accessed on 16 March 2022); China National Intellectual Property Administration (https://www.cnipa.gov.cn/, accessed on 16 March 2022); China Patent Information Center (http://www.cnpat.com.cn, accessed on 17 March 2022). EPSDATA (https://www.epsnet.com.cn/index.html#/, Home, accessed on 17 March 2022).

## Author contributions

The empirical work and the manuscript's first draft were performed by DL. The methodology guidance and software supporting were provided by FR. The conceptualization and funding supporting were provided by YL. All authors contributed the conception and design of this study. All authors read and approved the final manuscript.

## Funding

This work was supported by Philosophy and Social Science Fund project of Hunan Province [21YBQ079].

## Conflict of interest

The authors declare that the research was conducted in the absence of any commercial or financial relationships that could be construed as a potential conflict of interest.

## Publisher's note

All claims expressed in this article are solely those of the authors and do not necessarily represent those of their affiliated organizations, or those of the publisher, the editors and the reviewers. Any product that may be evaluated in this article, or claim that may be made by its manufacturer, is not guaranteed or endorsed by the publisher.
